# Comparative analysis of soil quality and enzymatic activities under different tillage based nutrient management practices in soybean–wheat cropping sequence in Vertisols

**DOI:** 10.1038/s41598-024-54512-z

**Published:** 2024-03-21

**Authors:** Satya Narayan Meena, Shanti Kumar Sharma, Pratap Singh, Bharat Prakash Meena, Asha Ram, Roshan Lal Meena, Deepak Singh, Ram Bhawan Meena, Mahaveer Nogiya, Devendra Jain, Kuldeep Kumar

**Affiliations:** 1 Agriculture University, Kota, Rajasthan 324001 India; 2 Indian Council of Agricultural Research (ICAR), New Delhi, 110012 India; 3 ICAR-Indian Institute of Soil Science, Bhopal, Madhya Pradesh 462 038 India; 4 ICAR-Central Agroforestry Research Institute, Jhansi, Uttar Pradesh 284003 India; 5 ICAR-National Bureau of Soil Survey and Land Use Planning, Regional Centre, Udaipur, Rajasthan 313 001 India; 6 ICAR-Indian Agricultural Statistical Research Institute, New Delhi, 110012 India; 7 ICAR-Indian Institute of Soil and Water Conservation, Research Centre, Agra, Uttar Pradesh 282 006 India; 8 Maharana Pratap University of Agriculture and Technology, Udaipur, Rajasthan 313004 India; 9 ICAR-Indian Institute of Soil and Water Conservation, Research Centre, Kota, Rajasthan 313004 India

**Keywords:** Conservation agriculture, Organic, Soil properties, Soybean, System productivity, Wheat, Ecology, Environmental sciences

## Abstract

In the modern era, intensive agricultural practices such as agrochemicals are applied in excessive amounts to enhance agricultural production. However, imbalanced adoption of these chemicals has arisen in the dwindling of agriculture factor productivity and soil quality. To maintain soil fertility and production, these chemical fertilizers must be supplemented with organic inputs. Keeping this in the backdrop, a research trail was established during 2018–19 and 2019–20 years at Research Farm of Agriculture University, Kota, India. The treatment setup was comprised of 5 treatment modules viz., conservation tillage + organic management (CAOM), conservation tillage + chemical management (CACM), conventional tillage + chemical management (CTCM), conventional tillage + organic management (CTOM) and the package of practices (PoPs) with four replications. Results indicated that the highest organic carbon (0.68%), bacterial (29.11 × 10^7^ cfu g^−1^), fungal (4.77 × 10^4^ cfu g^−1^), actinomycetes populations (5.67 × 10^4^ cfu g^−1^), acid phosphatase (44.1 µg g^−1^ h^−1^), urease (45.3 µg g^−1^ h^−1^) and dehydrogenase (23.3 µg triphenylformazan [TPF] g^−1^ h^−1^) activity in soil were found in the treatment of conservation organic system during both the years of study at each soil depth. In contrast to other parameters, the highest system productivity was observed with conservation chemical crop management approaches, with a soybean equivalent yield of 4615 kg ha^−1^ in a soybean–wheat system of production. Furthermore, the soil quality index (SQI) significantly varied from the lowest score (0.30) at 45–60 cm layer of soil in the package of practices to the highest score (0.92) at 0–15 cm layer of soil with regards to the conservation organic which shows, 206.67 percent enhancement through the soil profile of various crop management practices. The SQI variation from 0–15 to 45–60 cm soil depth was 130.0, 81.08, 60.0, 175.0 and 83.33 percent, respectively, for CAOM, CACM, CTCM, CTOM and PoPs. Amongst, different systems, the highest mean performance was noticed under the conservation organic systems for physical and biological properties. Hence, in line with the salient outcome, we may propose that the conservation chemical system needs to be followed to improve crop productivity, whereas, conservation organic seems a good option for soil health with long-term viability.

## Introduction

Large-scale adoption of high-yielding, nutrient-responsive cultivars, chemical fertilizers, improved irrigation and crop management practices and farm mechanization have enhanced the food grain production of India from 176.39 million metric tons (in 1991) to 316.0 million tons (in 2021–22)^1,2^. However, resource-intensive agricultural production systems have caused second-generation concerns such deteriorating factor productivity, resource consumption efficiency, soil quality, and farm profit. Long-term research experiments have shown that main food grain yields stagnate, decline, or respond poorly to fertilizers^3–7^. Further, there are more than 30 cropping systems in India^8^, and soybean is the main oilseed crop, grown on 12.81 million ha with 12.90 million tons production^9^. Its strong yields might boost oil seed produce in the country. Due to their N-fixation ability and soil fertility benefits, soybean farmers use little or no chemical fertilizers^10^. Moreover, the succeeding wheat crop has the carryover effect of the soybean crop^11^. Wheat, India's second-largest cereal crop after rice, covers 31.61 million hectares and produces 109.52 million tons. It covers 24.44% of food grain land and 35.48% of grain production^9^. In any agricultural system, soybeans and wheat are complimentary crops. There is a wide discrepancy in soybean and wheat potential with mean yields of 2.50 and 0.95 tones ha-1, 6.20 and 2.49, respectively^12^. Soybean and wheat needed balanced fertilization to maximize production. After the success of conservation agriculture under paddy-wheat^13,14^ and kharif maize-wheat^15–17^ cropping systems and increased awareness of organic foodgrains^18^, Indian farmers now use conventional, conservation, and organic agriculture. These crop-specific strategies impact crop growth, productivity, soil health, and economics^19^. Finding out how management approaches affect different crops and cropping systems is crucial to achieving high food production and comparing crop management practices under different cropping systems.

Conventional tillage practices have adversely affected soil quality and crop production both in sole crop and cropping systems^20,21^. Zero tillage, high plant residue and cropping pattern leads to improved crop yield and maintained soil health^22^. In zero-till farming, and soil environment is usually greater as compared to conventionally tilled soils^23^. Many other benefits of conservation agriculture are minimizing losses in terms of nutrients^24,25^, soil water, and also minimizing the implications of climate change on agricultural yield^26,27^.

Inorganic fertilizers without the insertion of biological sources for nutrients affect the soil properties and induce environmental contamination^28^. Utilizing biological sources, either separately or in conjunction to inorganic source of nutrients, will help to enhance soil qualities^29–31^. Organic manures maintain a favorable nutritional balance and provide better nutrition for the growth of soil organisms in order to achieve sustainable crop production^32^. Using fertilizers wisely in conjunction with organic resources can help preserve soil fertility for the sustainable production of soybean^33^. However, using only organic manures is insufficient to provide the crop's nutrient needs during the crop growth phase^34^. Additionally, it has been found that the micronutrient requirements of soybean are met by using organic manures in conjunction with fertilizers^35–37^.

Effective agriculture requires the judicious utilization of available resources as soils can lose their producing capacity within a short period time due to several reasons^38^. Best soil management practices are subject to several factors such as the availability of nutrient inputs, agronomic management practices, and also climatic factors^39^. Because of this, there has been a recent increase in attentiveness in assessing our soil resource's quality. Soil is a vital part of the earth's biosphere, serving not solely to produce food and fibre but additionally for sustaining local, regional, and global environmental quality^40^. The dependability and sustainability of management inputs will increase with decision tools and tactics that can help organize soil tests and evaluate how soil management activities affect soil ecosystems^41^. Soil quality indices (SQIs) are assessment tools that reliably integrate a variety of inputs for use in making multi-objective decisions^42^. Therefore, information is very limited to corroborate SQI versus region specific crop production as most of the scientific studies are concentrated as an ending variable, the environmental aspects of the soil but SQI for different soil layers has not been determined. However, evaluating SQIs against varied crop yields is difficult since SQIs calculated simply on surface soils may not reflect the true relationship between soil quality and yield because crop root systems may expand to deeper soil layers. Hence, in this study, the SQI was determined for multiple soil depths to investigate its true association with crop yields as well as other inter-dependent quality parameters.

As such the farming community is more concerned with crop productivity^43^ and monetary returns as compared to environmental quality^37,44^. To fulfill the needs of the expanding population for food, fibre, fuel, fodder, and other items, the productivity of farm land and the wellness of soil must be increased^45,46^. Numerous researches propound that, strategies for managing nutrients should change from resource-depleting chemical agriculture to resource-protecting organic or conservation agriculture^4,32,47^. Various work on organic as well as conservation agriculture are available for cropping systems in India that are focused on rice and maize, but only very limited information is available on soybean based copping systems. Keeping these things in mind, a field study was evaluated to find out the effect of tillage, organic and chemical management operations on soil enzymes, quality and chemical composition of soil and system production in the Vertisols of typical sub-tropical climatic conditions which nourish soybean–wheat system.

## Materials and methods

### Details of study site, climate and soil

**Figure 1:**
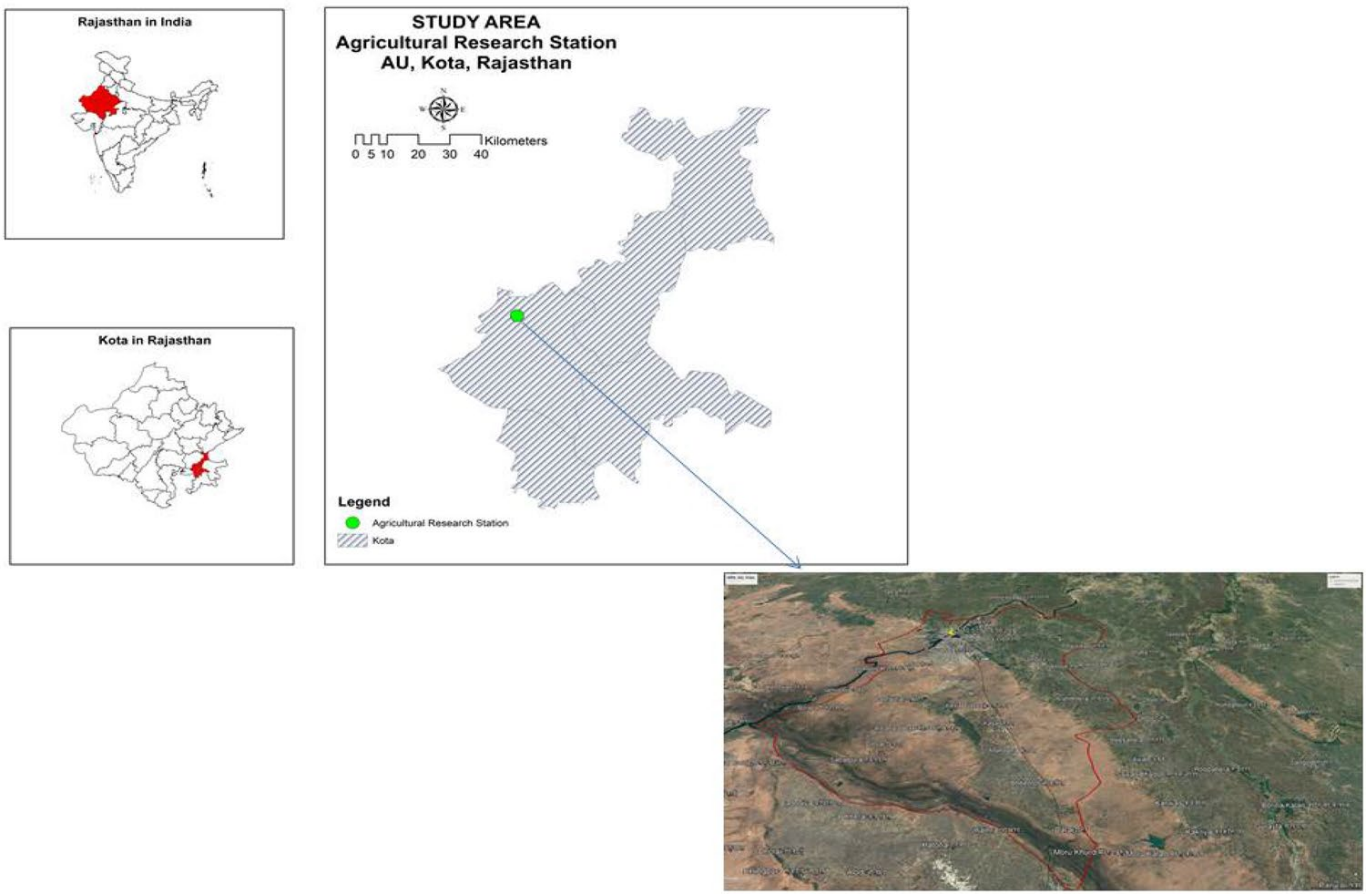
Location of the study area at Rajasthan Agriculture University, Kota, India.

The research work was investigated as the *kharif* and *rabi* sowing of 2018 and 2019 at Agricultural Research Station, (25° 10′ 57'' North Latitude; 75°50′ 20'' East Longitude and 267 m above MSL) of Agriculture University, Kota, Rajasthan, India (Fig. 1). The climatic condition of the region belongs to sub-tropical and is marked by mild winters and moderately long summers (hot and dry from late March to end of June). The average temperature during summer (May–June) ranges between 40.0 to 48.0 °C and 4.0 to 15.0 °C during winter (December-January). The study location’s average annual rainfall is 660 mm, majority of that occurring in monsoon season (June to September). The field of experimentation featured well-drained, fairly deep, black clay-loam Vertisols, comprising 25.86% sand, 35.10% silt, and 38.94% clay. The soil exhibited a slightly alkaline reaction with pH of 7.41. The BD (Mg m^−3^), porosity (%), SOC (g kg^-1^) and EC (dS m^–1^) of the upper surface (0–15 cm) of soil were 1.28, 51.0, 5.10 and 0.65, consequently. The experimental plots revealed a deficit in available nitrogen, a moderate level of available phosphorus, and an abundance of exchangeable potassium to the tune of 234.0, 21.13 and 440 kg ha^−1^, respectively. Depth wise details of initial soil properties are stated in Table 1.

**Table 1:** Soil properties at different depths of the experimental field (before *Kharif* 2018).

Soil parameters	Value				Method of analysis
	0–15 cm	15–30 cm	30–45 cm	45–60 cm	
A. Soil chemical parameters					
pH (1: 2.5 soil: water)	7.41	7.48	7.40	7.49	pH meter^48^
EC (dS m^−1^ at 25° C)	0.65	0.67	0.65	0.69	Conductivity bridge^49^
OC (%)	0.51	0.49	0.42	0.37	Walkley and Black^50^
B. Soil available nutrients					
N (kg ha^–1^)	234	210	199	190	Alkaline permanganate method^51^
P (kg ha^–1^)	21.13	19.95	18.00	17.57	Olsen’s P method^52^
K (kg ha^–1^)	440	426	418	411	Ammonium Acetate Method^53^
S (kg ha^–1^)	16.72	15.43	13.30	12.81	Turbidimetric procedure^54^
C. Soil biological parameters					
Total Bacteria (10^7^ × *cfu* g^−1^)	3.23	1.01	0.68	0.01	Standard serial dilution and plate count method^55^
Total Fungal (10^4^ × *cfu* g^−1^)	0.67	0.22	0.16	0.05	
Actinomycetes (10^4^ × *cfu* g^−1^)	0.38	0.31	0.19	0.07	
D. Enzymic Activities					
Acid phosphatase (µg P-nitrophenol g^−1^ h^−1^)	13.24	7.81	4.13	2.61	*P-nitrophenol* method^56^
Urease (µg g^−1^ h^−1^)	25.48	17.82	9.67	4.52	Tabatabai 1994^57^
Dehydrogenase (µg TPF g^−1^ h^−1^)	10.61	6.12	3.28	1.97	Tri-phenyl formazon^58^

### Experimental design and treatment details

The field demonstration was set down into RBD which includes 4 replications and 5 treatments viz., T1-conservation tillage + organic management (CAOM), T2-conservation tillage + chemical management (CACM), T3-conventional tillage + chemical management (CTCM), T4-conventional tillage + organic management (CTOM) and the T5-package of practices (PoPs). Table 2 lists the specifics of the treatments and their indications.

**Table 2:** Tillage, weed, and nutrient management specifics during experimentation.

	Season/crop	T_1_: Conservation tillage + organic management	T_2_: Conservation tillage + chemical management	T_3_: Conventional tillage + chemical management	T_4_: Conventional tillage + organic management	T_5:_ Package of practices
Tillage	*Kharif*/Soybean	One ploughing and direct sowing through seed drill, previous crop biomass retention (wheat @ 2.5 t ha^-1^)	Same as in T_1_	One summer ploughing, two ploughing with planking and sowing through seed drill	Same as in T_3_	Same as in T_3_
	*Rabi*/Wheat	One ploughing and direct sowing through seed drill previous crop biomass retention (soybean 1.5 t ha^-1^)	Same as in T_1_	three ploughing, sowing of wheat was completed through seed drill	Same as in T_3_	Same as in T_3_
Weed management	*Kharif*/Soybean	Non-chemical methods *i.e*., dust mulch at 20 DAS under cultural method and hand weeding at 35 DAS under mechanical method	Chemical method: a ready mixed herbicide viz. sodium acilfluorfen 16.5% + clodinafop propargyl 8% EC (165 + 80 g *a.i.* ha^-1^) as sprayed at 25 DAS in soybean	Same as in T_2_	Same as in T_1_	Same as in T_2_
	*Rabi*/Wheat	Non-chemical methods i.e., dust mulch at 20 DAS under cultural method and hand weeding at 35 DAS under mechanical method	Chemical method: a ready mixed herbicide viz. clodinafop-propargyl 15% WP + metsulfuron methyl 1% WP (48 + 4 g *a.i.* ha^-1^) was sprayed as PE at 32 DAS in wheat crop	Same as in T_2_	Same as in T_1_	Same as in T_2_
Nutrient management	*Kharif*/Soybean	Organic source N:P: K, 30:40:40 FYM 6 t ha^-1^ + PSB 600 g ha^-1^	FYM + Fertilizer N:P: K, 30:40:40 FYM 5 t ha^-1^ + (Urea 11 + SSP 200 + MOP 25 kg ha^-1^)	FYM + Fertilizer N:P:K, 30:40:40 FYM 5 t ha^-1^ + (Urea 11 + SSP 200 + MOP 25 kg ha^-1^)	Organic source N:P:K:S, 30:40:40:30 FYM 6 t ha^-1^ + PSB 600 g ha^-1^	FYM + N:P:K:S 20:40:40:30 FYM 10 t ha^-1^ + (Urea 65 + SSP 250 + MOP 67 + Elemental S 2.2 kg ha^-1^)
	*Rabi*/Wheat	Organic source N:P:K 180:40:30 FYM 36 t ha^-1^ + PSB 600 g ha^-1^	FYM + Fertilizer N:P:K 180:40:30 FYM 5 t ha^-1^ + PSB 600 g ha^-1^ + (Urea 337 + SSP 250 + MOP 50 kg ha^-1^)	FYM + Fertilizer N:P:K 180:40:30 FYM 5 t + PSB 600 g + (Urea 337 + SSP 250 + MOP 50 kg ha^-1^)	Organic source N:P:K 180:40:30 FYM 36 t ha^-1^ + PSB 600 g ha^−1^	Fertilizer N:P:K:Zn 120:40:30:25 PSB 600 g ha^−1^ + (Urea 260.9 + SSP 250 + MOP 50 + ZnSO_4._ 7H_2_O 25 kg ha^−1^)

*T_5_—Incorporation of FYM @ 10 t ha^−1^ for package of practices once in three years thus, applied only during experiment initiation *kharif* 2018.

#### Manure and fertilizer application

After field preparation, well-decomposed FYM was incorporated as per treatment details before sowing of the crops during each year of study. The amount of FYM was calculated as per N content and N requirement of the treatments. The nutrient concentration of farmyard manure (FYM) was examined for calculating total N adopting the Kjeldahl digestion procedure^48^ although phosphorus and potassium were analyzed adopting a wet digestion procedure^59^. Micronutrients in FYM were estimated by the diethylenetriaminepentaacetic acid (DTPA)-extract procedure using Atomic Absorption Spectrophotometer (AAS)^60^. The average nutrient composition of FYM was determined as shown: Nitrogen (0.50%), Phosphorus (0.26%), Potassium (0.50%), Sulphur (0.03%), Zinc (24.8 ppm), Iron (173.9 ppm), Copper (5.15 ppm), and Manganese (97.5 ppm).

### Crop management

Table lists the crop management techniques used for the wheat and soybean crops grown during the study period. The soybean (var. RKS 45) was sown @ 80 kg ha^-1^ seed rate through a seed drill around 15th July (with a row-to-row space of 30 cm) and harvested during 4th week of October. After the soybean harvest, pre-sowing irrigation was given for preparing the field and seeding of subsequent wheat crop. The wheat (var. Raj 4079) was sown during the 1st week of December (with an inter-row space of 22.5 cm and 100 kg ha^-1^ seed rate) and harvested in the first fortnight of April. The intercultural operations were performed according to the treatment plan outlined in Table 2. In the *kharif* season of 2018, single lifesaving irrigation was given at pod filling stage to soybean. However, in *kharif* season of 2019, the crop was grown without any irrigation due to sufficient moisture availability. For the *rabi* season, the wheat crop received a total of four irrigations during its critical growth phases. Inorganic fertilizers were applied according to the designated treatments. To soybean, the complete amount of N, P_2_O_5_, K_2_O, and S was applied at the time of sowing. Conversely, for wheat, 100% of P_2_O_5_, K_2_O, and ZnSO_4_, along with half of N, were used at planting time. The remaining 50% of nitrogen (N) was added during the initial watering event.

### Collection of soil samples

Soil samples were collected through a soil auger from 4 soil layers (0–15, 15–30, 30–45, and 45–60 cm) from each plot after each cropping season and arranged as a composite sample as per standard protocol for laboratory analysis of soil.

### Soil analysis

The key methods of soil properties assessment were followed as per Table 1. The experimental site's bulk density was assessed using a core sampler^61^. Soil porosity was subsequently calculated using the bulk density (BD) and particle density (PD) data, employing the formula below:

$$ {\text{Porosity}}(\% ) = \left( {1 - \frac{{{\text{Bulk}}\;{\text{Density}}}}{{{\text{Particle}}\;{\text{Density}}}}} \right) \times 100 $$

Biological properties viz*.,* total fungal, bacteria, and actinomycetes count, dehydrogenase activity (DHA) of different soil depth, and acid phosphatase was determined as per the standard protocols quoted in Table 1.

### Determination of soil quality indices

Soil quality indices (SQI) were assessed using standard method, which consisted of three main steps: Soil quality indicators selection, Normalization of indicator values into 0–1 scale, and Incorporation of transformed indicators into soil quality index^62^.

The intact analyzed data of various soil parameters beneath various treatments were subjected to principal component analysis (PCA), which has been extensively used to recognize the most sensitive SQ indicators^62,63^. Standardized PCA of untransformed (original observations) soil data was executed by the use of the Statistical Package for The Social Sciences (SPSS). The PCs that obtained eigenvalues ≥ 1 or at least explained ≥ 5% unevenness in the soil records were considered for qualifying the most sensitive soil quality indicator^64^. Variables with high weights were kept in a certain PC for the bare least data set of indicators. Here, the huge weights were indicated by absolute values that were inside 10% of the enormous factor loading^62,65^. Multivariate correlation coefficients were used to definitive whether many variables (such as soil parameters) should be considered redundant and if so, removed from the minimal set of information of indicators^66^. Each of the vastly weighted elements (soil variables) was deemed pertinent and kept to be comprised in the marginal data set of indicators if they were not well associated. Among soil variables with great correlation, only the variable with the highest weight factors (absolute) should be considered.

Each soil characteristic was first altered into a score (unit less), stretch from 0 to 1, by applying the linear scoring technique, after choosing the most relevant indicators for the least data set from among the various soil variables. In this procedure, groupings of selected soil indicators were created based on whether a larger value was beneficial or negative for soil function. In general, three mathematical method functions were utilized for this task: 1. More is better, 2. Less is better, and 3. Optimum is better. The best qualities are those that have a fortunate effect up to a predetermined level and may be regarded as harmful over that level. Scores range from 0 to 1, with 1 being the highest possible function for the system that was chosen.

The selected soil quality indicators were transformed into unitless values (scored 0–1) before being assimilated into soil quality indices for management practices using a biased additive indexing tactic. In this approach, the weightage was given to each observation of the minimum dataset variables using the PCA results. Every principal component (PC) explains a given extent of variation in the data, which was broken down by the total sum of all PCs (cumulative) used for the MDS of soil quality indicators to provide a specific weight under each PC. Then, the soil quality index (SQI) was calculated by the equation mentioned below:

$$ {\text{Soil}}\;{\text{Quality}}\;{\text{ Index }}\;\left( {{\text{SQI}}} \right) = \sum\limits_{{{\text{i}} = 1}}^{{\text{n}}} {{\text{Wi}} \times {\text{Si}}} $$

where S = score of the subscripted indicator, W = weightage derived from PCA results.

Here, the hypothesis is that higher SQI scores meant better soil quality or greater performance of the soil function.

### Crop yield and system productivity

In the experiment, wheat and soybean crops were hand-reaped for economic yield from net plots 20 cm above the ground. After five days of solar drying in the field, the reaped crops were bundled up and brought for the threshing. To estimate the total yield (economic + straw), the bundle weight of each crop or plot was recorded using a portable weighing balance. As soybean and wheat had distinct minimum support prices (MSPs), the system productivity was computed by transforming wheat yields into soybean equivalent yields (SEYs) using the formula below:

System productivity was determined by combining the soybean yield with the calculated equivalent soybean yield of the wheat for the respective years.

Minimum support price (MSP) of soybean (₹ 34,000 tonne^-1^ and ₹ 37,000 tonne^-1^) wheat (₹18,400 tonne^-1^ and ₹ 19,250 tonne^-1^) was used for calculating the soybean equivalent yield (GoI) during 2018–19 and 2019–20, respectively.

### Statistical data analysis

The impact of distinct treatments on soil characteristics (averaged across various depths ranging from 0 to 60 cm at 15 cm intervals) was examined using PCA biplot analysis. Additionally, a multivariate stability statistic was computed through the PCA biplot method with the assistance of RStudio. Within this ongoing research, we investigated the relationships between different treatment scenarios and variables. These variables were then grouped based on their stability and mean values^67^.

### Ethical approval

Soybean var. RKS 45 released by MPUAT, Udaipur (Rajasthan) and Wheat variety Raj 4079 was released by the RARI, Durgapura for the Rajasthan conditions. Both the varieties are commercially available in the public domain. Use of both the varieties in the present study complies with International, Indian, and/or institutional (herein MPUAT Udaipur and RARI, Durgapura) guidelines.

## Results

### Soil biological properties

#### Bacteria count

**Figure 2:**
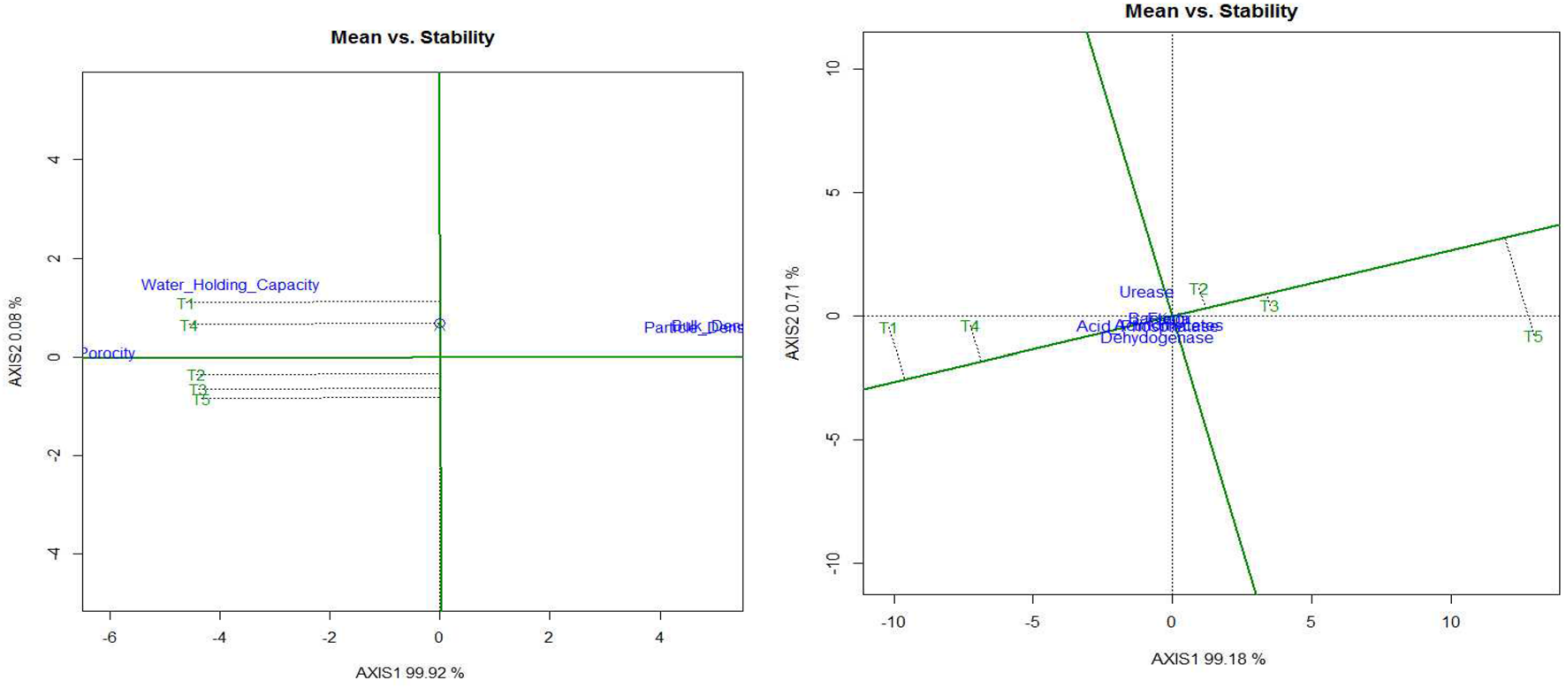
The mean versus stability perspective of treatment effects on soil biological and physical characteristics (biplot). The abbreviation section provides explanations for the labels used to represent the treatments.

**Figure 3:**
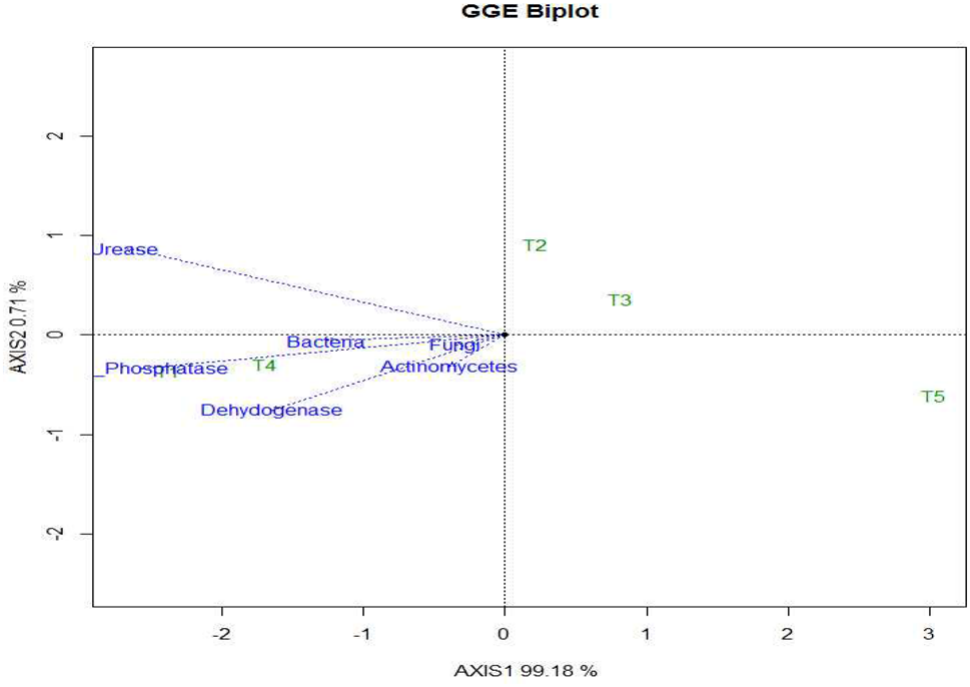
Treatment effects plus treatment × environment interaction effect (GGE) biplot of soil biological properties. The abbreviation section provides explanations for the labels used to represent the treatments.

Data summarized in Table 3, Figs. 2 and 3, a significantly higher soil bacteria population (29.11 × 10^7^) was recorded in conservation tillage with organic management in contrast to else treatments except for conventional tillage + organic management in top 0–15 cm soil. Incorporation of the organic management irrespective of the tillage treatments significantly increased the bacterial population. The standard package of practices (PoP) was recorded with almost 50% less bacteria population (14.25 × 10^7^) than conservation tillage with organic management at top soil of 0–15 cm. Though, regardless of the treatments, the population of bacteria significantly increased over the initial value (3.23 × 10^7^) after a two-year soybean–wheat cropping system. Similar trends were recorded at other soil depths (15–30, 15–45, and 45–60 cm). However, parallel to the surface soil, the bacterial population drastically diminished under lower soil depths.

**Table 3:** Effect of tillage based nutrient management techniques on total bacteria, total fungal count, and total actinomycetes at different soil depths (cm) (After *Rabi* 2019–20).

Treatments	Total bacteria (10^7^ × *cfu* g^−1^ soil)				Total fungal Count (10^4^ × *cfu* g^−1^ soil)				Total actinomycetes (10^4^ × *cfu* g^−1^ soil)			
	0–15	15–30	30–45	45–60	0–15	15–30	30–45	45–60	0–15	15–30	30–45	45–60
CAOM	29.11^a^	11.79^a^	7.28^a^	1.37^a^	4.77^a^	2.48^a^	1.15^a^	0.38^a^	5.67^a^	2.95^a^	1.08^a^	0.51^a^
CACM	21.58^b^	9.66^b^	3.84^c^	0.57^c^	2.97^b^	1.32^b^	0.48^c^	0.19^c^	2.93^b^	1.08^c^	0.58^c^	0.18^c^
CTCM	19.25^c^	8.58^c^	3.04^d^	0.30^d^	2.61^c^	0.98^c^	0.41^c^	0.13^d^	2.79^b^	0.89^d^	0.44^d^	0.16^c^
CTOM	28.18^a^	11.35^a^	6.04^b^	0.73^b^	4.56^a^	2.36^a^	1.01^b^	0.32^d^	5.38^a^	2.68^b^	0.89^b^	0.47^b^
PoPs	14.25^d^	4.53^d^	0.84^e^	0.08^e^	1.00^d^	0.46^d^	0.17^d^	0.03^e^	1.09^c^	0.83^d^	0.28^e^	0.03^d^

There is no significant difference between means with the same letter based on the DMRT (P = 0.05).

#### Fungi count

Organic management has not only increased the bacterial population but fungi population also. The fungal population within the uppermost 0–15 cm of soil increased significantly over earlier population (0.67 × 10^4^) with all the treatments. The conservation tillage with organic management recorded the highest fungal population (4.77 × 10^4^) followed by CTOM (4.56 × 10^4^), CACM (2.97 × 10^4^) and CTCM (2.61 × 10^4^). However, the lowest fungal population in the top 0–15 cm soil depth was proved while applying a package of practices (1.00 × 10^4^). Similarly, at soil layer of 15–30 cm significantly superior total population was registered with conservation tillage with organic management (2.48 × 10^4^) followed by conventional tillage with organic management (2.36 × 10^4^). A similar trend was observed with the other two depths (30-45 cm and 45–60 cm). Although, the fungal population subside drastically with soil depth but the effect of organic management reached at a lower depth also (Table 3, Figs. 2 and 3).

#### Actinomycetes count

Conservation tillage + organic management (CAOM) recorded a significantly higher actinomycetes population (5.67 × 10^4^) over other treatments but was found to be statistically non-significant with the CTOM (5.38 × 10^4^) at the uppermost 0–15 cm of the soil. The effect of organic management either with conservation or conventional, reached 0 to 60 cm soil depth. Through up to 60 cm soil depth, T1-CAOM and T4-CTOM were recorded with a comparatively higher (almost double) populations of actinomycetes than chemical management either with conservation or conventional tillage and the standard package of practices (Table 3 and Fig. 3).

#### Acid phosphatase activity

Different treatments of tillage, chemical and organic management significantly affected the acid phosphatase enzyme in the soil at different depths (Table 4, Figs. 2 and 3). Amongst all treatments, conservation tillage with organic management significantly increased activity of acid phosphatase enzyme followed by conventional tillage with organic management. The highest acid phosphatase activity (44.12 µg g^-1^ h^-1^) registered with CAOM and least with the package of practices (16.68 µg g^-1^ h^-1^) at top soil of 0–15 cm, after a two-year soybean–wheat cropping system. Similarly, at the soil of 15–30 cm, the higher activity of acid phosphatase was recorded with the application of CAOM (24.45 µg g^-1^ h^-1^) later by CTOM, CACM and CTCM to the tune of 22.56, 15.29, 12.35 µg g^-1^ h^-1^, respectively while, minimum values of acid phosphatase at 0–15 cm soil layer were found under PoPs (8.25 µg g^-1^ h^-1^). Furthermore, the result indicates that the concentration of acid phosphatase increased significantly in conservation tillage with organic management at lower depth *i.e.,* 30–45 cm and 45–60 cm to the tune of 11.57 and 5.81 µg g^-1^ h^-1^, respectively over the initial values.

**Table 4:** Effect of tillage based nutrient management practices on acid phosphatase, urease, and dehydrogenase at different soil depths (cm) (After *Rabi* 2019–20).

Treatments	Acid phosphatase (µg P-nitrophenol g^−1^ h^−1^)				Urease (µg g^−1^ h^−1^)				Dehydrogenase (DHA) (µg TPF g^−1^ h^−1^)			
	0–15	15–30	30–45	45–60	0–15	15–30	30–45	45–60	0–15	15–30	30–45	45–60
CAOM	44.12^a^	24.45^a^	11.57^a^	5.81^a^	45.28^a^	40.36^a^	18.17^a^	11.64^a^	23.31^a^	17.56^a^	9.78^a^	7.19^a^
CACM	30.25^c^	15.29^c^	7.63^c^	3.26^c^	37.65^b^	32.36^b^	14.88^b^	7.44^c^	14.12^b^	10.43^b^	8.05^b^	4.86^c^
CTCM	29.68^c^	12.35^d^	6.35^d^	2.99^c^	34.23^b^	28.67^b^	13.96^b^	7.23^c^	13.44^b^	10.27^b^	7.36^b^	4.25^d^
CTOM	39.22^b^	22.56^b^	9.90^b^	4.88^b^	42.26^a^	39.23^a^	16.96^a^	10.42^b^	22.36^a^	16.38^a^	9.42^a^	6.29^b^
PoPs	16.68^d^	8.25^e^	3.02^e^	1.76^d^	27.04^c^	16.29^c^	8.80^c^	4.83^d^	10.19^c^	7.42^c^	4.05^c^	1.89^e^

There is no significant difference between means with the same letter based on the DMRT (P = 0.05).

#### Urease enzyme activity

Urease concentration in soybean–wheat cropping systems grew notably over starting levels in all treatments. However, organic management with either of the tillage treatments, increased urease activity significantly over the other treatments (Table 4, Figs. 2 and 3). Conservation tillage with organic management stimulated urease activity (45.28 µg g^-1^ h^-1^) in soil followed by CTOM (42.26 µg g^-1^ h^-1^) at soil layer of 0–15 cm. An almost comparable pattern was detected under rest treatments at lower soil depths. Although urease activity was also reduced in lower soil depths but significant difference in various treatments were clearly observed.

#### Dehydrogenase activity (DHA)

Irrespective of the treatments, the activity of dehydrogenase increased significantly after the experiment on the soybean–wheat cropping system over its initial value. The treatment with conservation tillage + organic management registered superior dehydrogenase activity (23.31 µg TPF g^-1^ h^-1^) trailed by CTOM (22.6 µg TPF g^-1^ h^-1^). Both CAOM and CTOM statistically remained inconsequential to each other but notably admirable over the other treatments at dissimilar soil depths (Table 4, Figs. 2 and 3). However, the lowest activity of dehydrogenase at all the soil depths was recorded with the package of practice treatment. Although a notable reduction in dehydrogenase activity in all the treatments was observed at 45–60 cm soil depth but organic management (either with conventional or conservation) was recorded with more than 3 times higher activity as compared to the package of practice.

### Soil bulk density, particle density, and porosity

**Figure 4:**
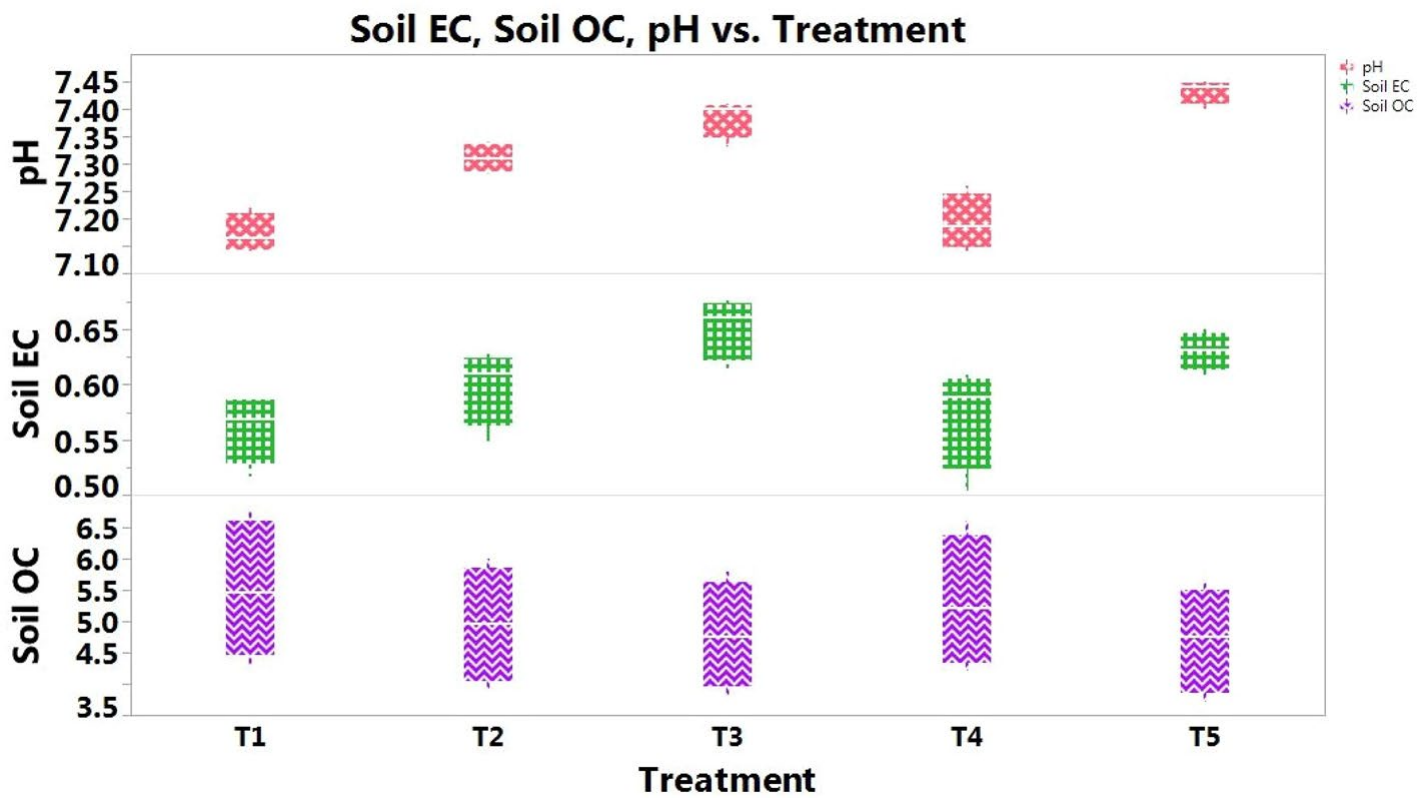
Soil EC (dS m^-1^), pH, and SOC (g kg^-1^ soil) (average of different soil depths) under different management scenario in soybean–wheat cropping system (After *Rabi* 2019–20).

Conservation, organic, and conventional crop management techniques had no discernible impact on the physical characteristics of the soil, viz*.,* BD, PD, soil porosity, and WHC, after two-year of study (Figs. 2 and 4). Although, CAOM crop management practices reduced BD**,** PD and improved soil porosity at the top 0–15 cm soil. The BD was also decreased by 0.03, 0.06, 0.08 and 0.10 g cm^-3^ with CAOM than CTOM, CACM, CTCM and package of practices, respectively. Similarly, soil particle density (PD) was reduced by 0.04, 0.06, 0.09 and 0.11 g cm^-3^ with CAOM over CTOM, CACM, CTCM and package of practices, respectively. Likewise, conservation tillage with organic management was recorded with higher porosity, but statistically, no differences were observed amongst all the treatments.

### Soil pH and EC

pH and EC of soil were registered as non-significant among all the treatments up to 60 cm at the depth interval of 15 cm during the experiment (Table 5 and Fig. 4). However, the supplication of crop residue and organic manures in CAOM reduced the pH of soil from 7.41, 7.48, 7.40, and 7.49 to 7.15, 7.14, 7.11 and 7.18 up from 0–60 cm at the depth interval of 15 cm, respectively. Likewise, soil EC decreased at different soil depth after the FYM and crop residue incorporation in conservation tillage + organic management treatment followed by conventional tillage + organic management as compared to other management crop management practices. However, all the treatments statistically remained at par with respect to soil pH and EC.

**Table 5:** Effect of tillage based nutrient management practices on soil pH, EC, and SOC at different soil depths (cm) (After *Rabi* 2019–20).

Treatments	Soil pH				Soil EC (dS m^−1^)				SOC (g kg^−1^ soil)			
	0–15	15–30	30–45	45–60	0–15	15–30	30–45	45–60	0–15	15–30	30–45	45–60
CAOM	7.15^a^	7.14^a^	7.11^a^	7.18^a^	0.52^a^	0.56^a^	0.56^a^	0.59^a^	6.8^a^	6.5^a^	4.9^a^	4.3^a^
CACM	7.28^a^	7.32^a^	7.29^a^	7.34^a^	0.55^a^	0.60^a^	0.60^a^	0.63^a^	6.0^b^	5.6^c^	4.5^bc^	3.9^b^
CTCM	7.33^a^	7.40^a^	7.37^a^	7.41^a^	0.61^a^	0.66^a^	0.66^a^	0.68^a^	5.8^bc^	5.2^d^	4.4^c^	3.8^b^
CTOM	7.14^a^	7.17^a^	7.19^a^	7.26^a^	0.50^a^	0.58^a^	0.58^a^	0.61^a^	6.6^a^	6.0^b^	4.7^ab^	4.2^a^
PoPs	7.40^a^	7.44^a^	7.43^a^	7.45^a^	0.61^a^	0.63^a^	0.63^a^	0.65^a^	5.6^c^	5.0^d^	4.3^c^	3.7^c^

There is no significant difference between means with the same letter based on the DMRT (P = 0.05).

### Organic carbon

The data showed that after two-year of soybean–wheat cropping systems, soil organic carbon increased significantly under conservation tillage + organic management (Table 5 and Fig. 4). Compared to CACM, CTCM and package of practices (PoPs), 13.33, 17.24, 21.42% higher SOC was documented respectively in the CAOM scenario. However, SOC content at top soil 0–15 cm depth under CTOM was also found at par with CAOM. Under 15–30 cm soil depth, the difference between various crop management practices was significant but the average SOC in the soil was decreased by 4.41 to 26.47% compared to soil depth of 0–15 cm. The incorporation of organic matter not only enhanced the SOC in the uppermost soil but also brought a significant effect on SOC in deeper soil depths. However, both the organic management treatments (conservation as well as conventional) remained non-significant to each other and notably higher over other management scenarios.

### Mean performance vs stability of the treatments and genetics, genetics × environment (GGE) biplot analysis

The PCA biplot analysis, illustrating the relationship between mean and stability, provided a comprehensive understanding of soil physical properties' response to treatment (100% variance explained) as well as treatment versus environmental variations. Meanwhile, soil biological properties accounted for 99.89% of the total variance. In Fig. 2, the average environment coordinate (AEC) exhibited a single direction, with the arrow indicating the higher-category outcome on a unique partitioning value (SVP = 1). Clearly, the superior mean show of soil physical properties was recorded under conservation tillage + organic management followed by conventional tillage + organic management. In case of soil biological properties (bacteria, fungi, actinomycetes, acid phosphatase, urease and dehydrogenase), the higher mean value was observed with conservation tillage + organic management followed by conventional tillage + organic management with moderate stability and lowest mean with package of practices with very high stability (Fig. 2). In PCA biplot analysis (GGE biplot) of soil microbial properties indicated microbial populace and soil enzymatic actions improved significantly under CAOM and CACM only.

### Soil quality index

The statistics on the presentation of SQ indicators in relation to factor loading (eigenvector) standards in PCA under different crop managing activities are mentioned in Table 6. In the present experiment, we considered the soil parameters with principal components (PCs) having an eigenvalue of ≥ 1 or which explicated tiniest ≥ 5% of the deviation in the soil parameters. The amount of swing explained by PC-1, PC-2, PC-3, PC-4, and PC-5, was 58.12, 11.28, 10.28, 9.65, and 6.84 percent, respectively. The vastly weighted variables (having outright amounts inside 10 percent of the maximum factor loading under the same PC) inside each PC were recalled to include in the tiniest data set (MDS). Hence, the bold-face tenets [Table 6, Bacteria (0.891), Fungi (0.896), Actinomycetes (0.916), Acid phosphatase (0.935), Urease (0.877), and DHA (0.938) in PC-1, EC (-0.864) in PC-2, available-P (0.842) in PC-3, SOC (0.958) in PC-4 and available-N (0.608) in PC-5] were found highly weighted factor loading. Accordingly, boldfaced variables were initially selected in the minimum data set. However, The PC-1 retained more than one variable as a high loading factor, but all these variables were found greatly correspond with one another in the inter-correlation study (Table 7). Thus, following the well-correlated criteria, the DHA in PC-1 was kept in the MDS, and others were terminated due to a high and significant correlation with retained variables in the PC. Though, in the PC-2, PC-3, PC-4 and PC-5 only sole variables viz. EC, available-P, SOC, and available-N were competent for MDS, respectively. So, from PC-1 to PC-5, DHA, EC, available-P, SOC, and available-N were reserved for final MDS to develop SQ indices under different management practices.

**Table 6:** Results of PCA of various SQ indicators.

	PC1	PC2	PC3	PC4	PC5
Eigenvalues	6.97	1.35	1.23	1.16	0.82
% Variance	58.12	11.28	10.28	9.65	6.84
Cumulative variance	58.12	69.40	79.68	89.33	96.17
Eigen vectors or factor loading					
Bacteria	**0.891**	0.258	0.189	− 0.071	0.222
Fungi	**0.896**	0.296	0.213	− 0.169	0.094
Actinomycetes	**0.916**	0.117	0.256	− 0.16	0.098
pH	0.827	0.191	0.277	− 0.100	0.375
EC	− 0.284	− **0.864**	− 0.197	0.310	− 0.068
Acid phosphatase	**0.935**	0.203	0.214	− 0.065	0.149
Urease	**0.877**	0.063	0.234	− 0.101	0.293
Dehydrogenase activity	**0.938**	0.248	0.183	− 0.114	0.002
SOC	− 0.121	− 0.224	− 0.103	**0.958**	− 0.051
Available-N	0.718	0.123	0.25	− 0.116	**0.608**
Available-P	0.433	0.241	**0.842**	− 0.153	0.145
Available-K	0.731	0.414	0.235	− 0.100	0.327

*Bold values under each principal component are highly weighted and underlined bold values are selected in the minimum data set for SQI determination.

**Table 7:** Correlation matrix (r) between highly weighted variables under PC-1.

PC-1 variables	Bacteria	Fungi	Actinomycetes	Acid phosphatase	Urease	DHA
Bacteria	1.0					
Fungi	0.954**	1.0				
Actinomycetes	0.937**	0.977**	1.0			
Acid phosphatase	0.972**	0.964**	0.973**	1.0		
Urease	0.889**	0.875**	0.877**	0.928**	1.0	
DHA	0.897**	0.926**	0.949**	0.950**	0.943**	1.0

**Correlation is significant at the 0.01 level (2-tailed).

In our results under different management practices, the “More is better” tactic was used for wholly screened soil quality indicators excluding soil EC picked up under PC-2, where the “less is better” task was used. The weighted factor acquire through the PCA findings was then multiplied by the scores obtained for each of the chosen indicators (Table 6). PC-1, PC-2, PC-3, PC-4, and PC-5 had weighted factors of 0.60, 0.18, 0.11, 0.10, and 0.07, respectively.

The results obtained under different management practices revealed that the SQ indices were affected significantly by various treatments and soil depths effects (Table 8). At soil depth of 0–15 cm, conservation tillage + organic management (0.92) and conventional tillage + organic management (0.88) recorded notably superior SQI over CACM (0.67) by 37.31 and 31.34 percent, over CTCM (0.64) by 43.75 and 37.50 percent and over the package of practices (0.55) by 67.27 and 60.0 percent, respectively. Further CACM and CTCM treatment also secured improved SQI over package of practices by 21.82 and 16.36 percent, respectively. At soil depth of 15–30 cm, treatment CAOM (0.75) recorded remarkably better SQI over CACM (0.56), CTCM (0.53), CTOM (0.70) and package of practices (0.47) by 33.93, 41.51, 7.14, and 59.57 percent, respectively. Further, CTOM also secured the better SQI over CACM, CTCM and package of practices by 25.0, 32.08 and 48.94 percent, respectively. At 30–45 cm soil depth, conservation tillage with organic management (CAOM) (0.50) and CTCM (0.50) noticed significantly higher SQI over CACM (0.47), CTOM (0.39) and package of practices (0.36) by 6.38, 28.21 and 38.89 percent, respectively. Further, CACM also recorded 20.51 and 30.56 percent superior SQI over CTOM and package of practices, respectively. SQI data of 45–60 cm soil depth also followed the same pattern as 30–45 cm soil depth. The SQI varied from the lowest value (0.30) at 45–60 cm soil layer under package of practices to the highest value (0.92) at 0–15 cm soil depth in the CAOM which shows, 206.67 percent, variation through the soil profile. The SQI variation from 0–15 to 45–60 was 130.0, 81.08, 60.0, 175.0 and 83.33 percent, for CAOM, CACM, CTCM, CTOM and PoPs, respectively.

**Table 8:** SQI under various treatments of crop management practices.

Treatments	Depth (cm)			
	0–15	15–30	30–45	45–60
CAOM	0.92^a^	0.75^a^	0.50^a^	0.40^a^
CACM	0.67^b^	0.56^c^	0.47^b^	0.37^b^
CTCM	0.64^b^	0.53^c^	0.50^a^	0.40^a^
CTOM	0.88^a^	0.70^b^	0.39^c^	0.32^c^
PoPs	0.55^c^	0.47^d^	0.36^c^	0.30^c^

There is no significant difference between means with the same letter based on the DMRT (P = 0.05).

### Crop yield and system productivity

**Figure 5:**
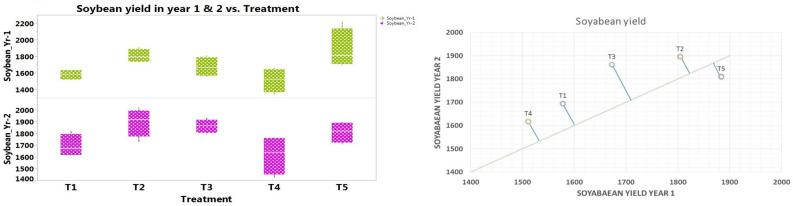
Effect of tillage and nutrient management on the soybean yield (kg ha^-1^) (*Kharif* 2018 & *Kharif* 2019).

**Figure 6:**
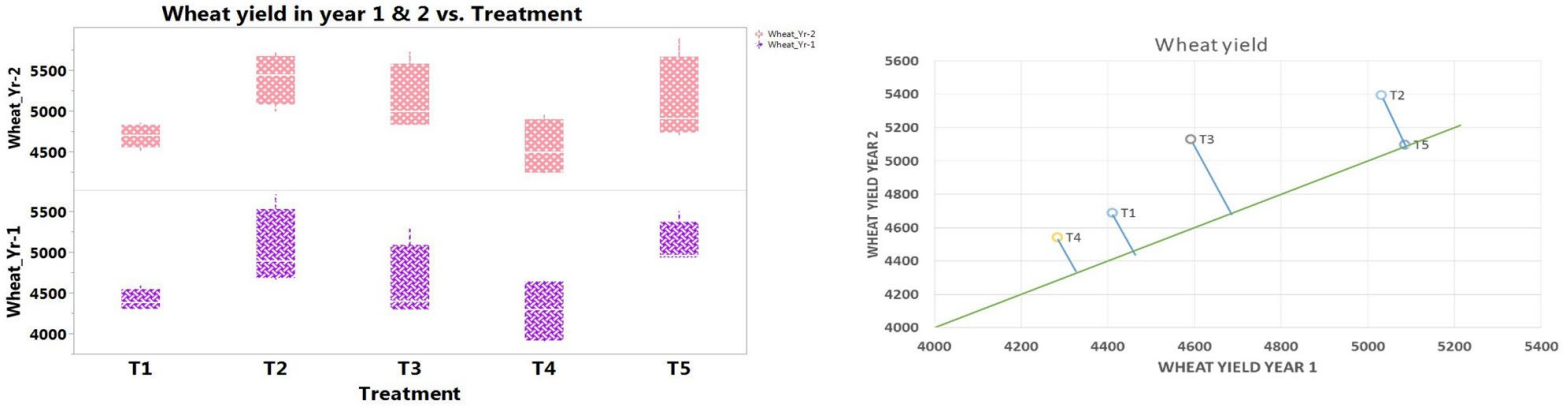
Effect of tillage and nutrient management on the wheat yield (kg/ha) (*Rabi* 2018–19* & Rabi* 2019–20).

Unlike nutrient availability and microbial properties, notably superior seed yield of soybean and wheat (pooled average 1850 and 5214 kg ha^-1^, respectively) was realized in CACM (T2) followed by the package of practices (T5) (Table 9, Figs. 5 and 6). However, in both the crops, three treatments (CACM, CTCM and package of practices) remained statistically non-significant to each other and notably superior over organic management treatments (T1 and T4). Compared to grain/seed yield of soybean and wheat in CTOM (T4), almost 18% higher yields were recorded in CACM in both the crops. Conservation tillage with chemical management (CTCM) secured notably superior system productivity of soybean–wheat cropping and it was notably superior by 13.96 and 18.17% over CAOM and CTOM, correspondingly (Table 9). Although, system productivity with CACM (T2) and package of practices (T5) was comparable with each other. It is clear that organic management (with conservation or conventional tillage) significantly enhanced soil accessibility to nutrients and microbial population, but system productivity was significantly lower than the chemical management and package of practices during the study.

**Table 9:** Effect of tillage based nutrient management practices on seed yields (kg ha^−1^) and system productivity of soybean–wheat system (pooled mean of 2 years).

Treatments	Soybean seed yield (kg ha^−1^)	Wheat grain yield (kg ha^−1^)	System productivity (SEY kg ha^−1^)
CAOM	1636^bc^	4550^b^	4049^c^
CACM	1850^a^	5214^a^	4615^a^
CTCM	1767^ab^	4861^ab^	4344^b^
CTOM	1564^c^	4413^b^	3905^c^
PoPs	1847^a^	5092^a^	4549^ab^

There is no significant difference between means with the same letter based on the DMRT (P = 0.05).

## Discussion

Organic management and conservation tillage practices culminated in a considerable escalate in the population of soil microbes including activities of the enzymes (Tables 3, 4, Figs. 2 and 3). Increased organic matter may cause higher microbial activities through providing energy and nutrients. Soil biological properties were comparatively lower in conventional tillage chemical management and package of practice in comparison to conservation tillage with organic management and conservation tillage with chemical management. Inorganic fertilizers enhanced crop productivity but due to lack of organic supplements it adversely affects the soil properties and health whereas, fertilizer application in association with organic matter help to enhance the action of microbes^68,69^. High organic deposit in the soils dispenses an increased biologically active carbon phase, which plays a key role for microorganism as an energy source. The activity of soil dehydrogenase and alkaline phosphatase are strongly regulated by soil organic carbon concentration. The higher enzyme activity is the outcome of applying organic manure and the addition of organic manure and soil enzyme activity are positively correlated^70–72^.

In comparison to CTCM treatments, organic farming increased the enzyme activity for twain dehydrogenase (98.2 μg TPF g^-1^ day^-1^) and alkaline phosphatase (178.2 g PNP g-12 h-1)^73^. The incorporation of organic manure amplifies the activity of enzymes and proved that an affirmative co-relationship pertains among them^74^. Soil parameters were drove by tillage based nutrient management practices in the soybean–wheat cropping sequence at different soil depths. Results indicated that conservation tillage and organic management slightly decrease BD and increase porosity and WHC of soil, but the effect was not significant (Fig. 2). This might be due to the implementation of minimum tillage practices, the addition of FYM^75^ and crop residue retention under these particular treatments. It is true that organic mediated treatments increase the porosity and WHC of the soil and lowers the BD and PD of the soil^32,76–78^, but the application of various management practices for only two years was not sufficient to bring about significant changes. Conservation tillage, including residue maintenance in the soil, helps to reduce BD. Due to its excellent buffering capacity; all treatments did not appreciably change soil pH and EC (higher clay content 38.94%)^79,80^.

The supply of organic nutrients and synthetic fertilizers for a short time (one-two years) had an inessential effect on soil pH^81^. PoPs may have higher soil EC values as a result of salts being added to the soil over time by synthetic fertilizers^82,83^. In contrast, a lower value of soil EC was recorded under organic management, which might be due to increased buffering capacity against salt accumulation^84,85^.

Conservation tillage organic management registered the highest SOC, followed by CTOM, CACM, and CTCM treatment (Table 5, Figs. 2, 3 and 4) in the different soil depth classes. Conservation tillage with organic management practices had a notable impact on soil properties in contrast to other practices. Better soil ecosystems for nutrient cycling and a higher supply of organic carbon from organic sources could be responsible for this^37,86^. Because of a favorable interacting effect on mineral N, N transformations can be converted by mixing quality residues with fertilizer N^87^. SOC was higher in the pulse-based rotation with no-tillage + crop residues than in the no residue treatments. A similar study also registered 105 and 71% superior SOC under long term experiments of organic farming as compared to control and recommended doses of NPK fertilizers, correspondingly^88^. One more study revealed that organic matter slowly accumulates in the soils where no/less disturbance is observed and diminishes with deep tillage treatments^89^. Application of FYM (20 Mgha^–1^ year^–1^) increased in organic management, and residue in conservation management increased the SOC concentration^30,31,90^. Positive impact of organic management on SOC and biological properties upto 60 cm soil depth may result from leaching effect of organic matter^91^. Higher growth of the plant and microbial populations further aggravates the availability of SOC^92–94^.

The soil quality aspects varied significantly under various management operations, and the treatments CAOM and CTOM practices had a higher soil quality index (SQI) (Table 8) than other treatments. The variations in SQIs in each treatment might be associated with the differences in management practices and organic as well as inorganic inputs. Organic matter supplies vital nutrients to soil, and conservation tillage reduces the losses of organic carbon in soil, which may stimulate microbe occupancy in the soil; therefore, the treatment with organic management and conservation tillage improved the SQI. The consistent addition of higher plant and root biomass and a lower degree of soil disturbance under conservation organic and organic management + conventional tillage practices improved the nutrient cycling, organic carbon content, and soil aggregation, which might be reflected in the form of higher SQI scores. Numerous aspects contributed to improving the SQI, like inputs of SOM through crop biomass, maximum biological and biochemical activity, fertilizer inputs, nutrient cycling, better soil aggregation, physical resistance, etc.^95^ and these all are represented by some of the most sensitive indicators. A study divulges that holistic use of lands with proper and suitable land management techniques is the most useful way to sustain and renovate ecosystem sustainability and soil quality through improving biomass of soil microbes, aggregate stability, soil respiration, and the biodiversity of fauna in the soil^96^. Long term cultivation with inappropriate land-use management and involvement of intensive tillage operations led to soil chemical impoverishment, higher soil compaction with its detrimental influences on soil physical properties, and negative effects on biological soil indicators driven by soil organic carbon reduction, as proved by the low SQI score in the agricultural land-use system^97^. The lower SQI under CTCM could be attributed to less organic biomass inputs incorporation into the soil and no accumulation of SOC. SQI values were noticed to be superior in upper soil layers than in lower soil layers, which could be due to most biological and organic matter accumulation occurred in surface soil. Maximum biological activity was observed up to a 30 cm soil depth, and soil microbial counts decreased as soil depth increased^97^. A study in Brazilian tropical conditions recorded decreasing trend of SQI with increasing soil depth^95^.

Yields of soybean and wheat and their system productivity (SEY) were notably affected by applied treatment, and higher crop productivity was recorded with CACM, followed by PoPs, CTCM, CAOM, and CTOM (Table 9, Figs. 5 and 6). Higher yields in conservation tillage with chemical management may be attributed to effective weed management and easy nutrient availability. Crop management techniques have an intense effect on soil characters and thereby on crop production individual as well as system yield^37,98^. Tillage practices, nutrient management, and the incorporation of leguminous crops all play a significant role in influencing crop yields within cropping systems^99,100^. A study highlighted that CACM (Conservation tillage with Crop Management) exhibited superior outcomes, resulting in the highest economic yield of soybean compared to alternative practices such as CTCM (Conventional Tillage with Chemical Management), CAOM (Conservation Agriculture with Organic Management), and CTOM (Conventional Tillage with Organic Management)^7,101^. In the wheat crop, similar findings were also reported^19^. In line with^102^ the inclusion of FYM (Farm Yard Manure) or crop liter conjointly with synthetic fertilizers was found to notably enhance the sustainability and consistency of productivity within the soybean–wheat system.

## Conclusion

This study demonstrated that organic management, whether conservation or conventional, increased soil fertility (SOC, soil quality index), microbial numbers, and enzymatic activity. Organic management and tillage did not change soil physical properties. However, conservation tillage and organic management modules recorded the most soil SOC, bacteria, fungi, actinomycetes, acid phosphatase, urease, and dehydrogenase. The same trend of nutrient contents, microbial population, and enzyme activities was also recorded at different soil layers. However, available soil nutrients and microbial activities decreased drastically with an increase in soil depth. The maximum crop yield and system productivity were recorded with conservation tillage chemical management modules. Furthermore, the PCA selected soil variables (DHA, EC, available-P, SOC, and available N) are sensitive to disruptions of the various agronomical management approaches, so attention should be paid to them. Thus, in the soybean–wheat cropping system, conservation tillage with chemical management may increase productivity, while conservation tillage with organic management promotes soil fitness and sustainability. A long-term study on the effects of conservation tillage with organic management approaches on soybean–wheat cropping system sustainability and production is possible.

## Data Availability

According to the institution's research policy, the research datasets created and/or analysed during this work are not publicly available, although data can be obtained from the corresponding author upon reasonable request.
